# Identification of Biomarkers Correlated with the TNM Staging and Overall Survival of Patients with Bladder Cancer

**DOI:** 10.3389/fphys.2017.00947

**Published:** 2017-11-28

**Authors:** Sheng Li, Xiaoping Liu, Tongzu Liu, Xiangyu Meng, Xiaohong Yin, Cheng Fang, Di Huang, Yue Cao, Hong Weng, Xiantao Zeng, Xinghuan Wang

**Affiliations:** ^1^Department of Urology, Zhongnan Hospital of Wuhan University, Wuhan, China; ^2^Department of Biological Repositories, Zhongnan Hospital of Wuhan University, Wuhan, China; ^3^Center for Evidence-Based and Translational Medicine, Zhongnan Hospital of Wuhan University, Wuhan, China

**Keywords:** bladder cancer, biomarkers, WGCNA

## Abstract

**Objective:** To identify candidate biomarkers correlated with clinical prognosis of patients with bladder cancer (BC).

**Methods:** Weighted gene co-expression network analysis was applied to build a co-expression network to identify hub genes correlated with tumor node metastasis (TNM) staging of BC patients. Functional enrichment analysis was conducted to functionally annotate the hub genes. Protein-protein interaction network analysis of hub genes was performed to identify the interactions among the hub genes. Survival analyses were conducted to characterize the role of hub genes on the survival of BC patients. Gene set enrichment analyses were conducted to find the potential mechanisms involved in the tumor proliferation promoted by hub genes.

**Results:** Based on the results of topological overlap measure based clustering and the inclusion criteria, top 50 hub genes were identified. Hub genes were enriched in cell proliferation associated gene ontology terms (mitotic sister chromatid segregation, mitotic cell cycle and, cell cycle, etc.) and Kyoto Encyclopedia of Genes and Genomes (KEGG) pathways (cell cycle, Oocyte meiosis, etc.). 17 hub genes were found to interact with ≥5 of the hub genes. Survival analysis of hub genes suggested that lower expression of MMP11, COL5A2, CDC25B, TOP2A, CENPF, CDCA3, TK1, TPX2, CDCA8, AEBP1, and FOXM1were associated with better overall survival of BC patients. BC samples with higher expression of hub genes were enriched in gene sets associated with P53 pathway, apical junction, mitotic spindle, G2M checkpoint, and myogenesis, etc.

**Conclusions:** We identified several candidate biomarkers correlated with the TNM staging and overall survival of BC patients. Accordingly, they might be used as potential diagnostic biomarkers and therapeutic targets with clinical utility.

## Introduction

Bladder cancer (BC) is the second most frequent genitourinary malignancy and the sixth most common malignancy in men. BC represents a spectrum of disease ranging from superficial, well-differentiated disease, which does not significantly affect the survival of BC patients, to highly fatal tumors for which long-term survival may be dismal (So, 2016; Ghervan et al., 2017). With the aging of population, the incidence of BC is rising year by year, and BC in elder patients will become even more frequent and evolved into a public health challenge in future. For patients with superficial BC, telescopic removal of the cancer (transurethral resection of bladder tumor, TURBT) followed by instillation of chemotherapy or vaccine-based therapy into the bladder with prolonged telescopic checking of the bladder are usually recommended, and the 5-year overall survival for these patients reaches 90%, while about 40–80% of these patients will develop disease recurrence or progression (Malmström et al., 2017). For patients with invasive BC, radical cystectomy plus pelvic lymph node dissection (PLND) followed by neo-adjuvant chemotherapy is recommended as a standard of care, and once it becomes metastatic cancer, the 5-year overall survival for patients with invasive BC is a dismal 6% (Salama et al., 2016; Sargos et al., 2016).

Biomarkers are biological substances whose detection indicates a particular disease state. So far, a variety of biomarkers have been introduced in daily clinical practice, including risk assessment, screening, differential diagnosis, determination of prognosis, prediction of response to treatment, and monitoring of progression of disease. Thus, identification of biomarkers that are associated with clinical outcomes of patients with BC might be of clinical significance (Giunchi et al., 2017).

Currently, several screening algorithms based on gene expression data, including Gene Set Enrichment Analysis (GSEA), Signalling Pathway Impact Analysis (SPIA), Topology Gene-Set Analysis, and DEGraph, and *in silico* Pathway Activation Network Decomposition Analysis (iPANDA), etc., have been introduced for both academic and commercial purpose, and most of these algorithms are intended to identify differentially expressed genes between groups of samples (Ozerov et al., 2016). Weighted gene co-expression network analysis (WGCNA), a systems biology algorithm, can be applied to describing the correlation patterns among genes across microarray samples, finding and summarizing modules of high related genes, and relating modules to certain clinical phenotype (Zhang and Horvath, 2005; Yip and Horvath, 2007). During the network construction, highly co-expressed genes are connected in the network and grouped into modules, thus, different modules included different functionally related genes and the most central and connected genes are treated as hub genes (Zhang and Horvath, 2005; Yip and Horvath, 2007). Correlation networks facilitate network based gene screening methods that can be used to identify candidate biomarkers or therapeutic targets. Therefore, WGCNA is usually used to facilitate the screening or identification of candidate biomarkers or therapeutic targets. Tumor node metastasis (TNM) 2009 (7th edition) was recommended for the BC, and previous studies demonstrated that patients with an infiltrative pattern had better survival than those with other pattern types (PDQ Adult Treatment Editorial Board, 2002; Yaxley, 2016). In the present study, the WGCNA algorithm was applied to identify candidate biomarkers for BC based on the TNM staging of BC patients.

## Methods

### Data sources and data preprocessing

Gene expression profile of GSE13507 (Kim et al., 2010; Lee et al., 2010) was downloaded from Gene Expression Omnibus (GEO) database. GSE13507 is a microarray dataset containing 165 primary BC samples, 23 recurrent non-muscle invasive tumor tissues, 58 normal looking bladder mucosae surrounding cancer and 10 normal bladder mucosae, the clinical characteristics of included samples are attached as well. In the present study, only the 165 primary BC samples were included for subsequent analysis. Preprocessed gene expression profile of GSE13507 was obtained from GEO database for our WGCNA analysis. Probesets were filtered by their variance across all samples, only probes with variances ranked in top 10,000 were selected for subsequent analyses.

### Co-expression network construction and detection of hub genes

The R package “WGCNA” (Langfelder and Horvath, 2008) was used to build co-expression network for the filtered gene expression matrix. Before building a co-expression network, we applied sample networks method introduced by Oldham et al for outlier detection. We designated sample as outlying, if the Z.K value was below −2.5. The soft threshold power β was selected based on the scale-free topology criterion introduced by Zhang and Horvath (2005). We calculated Pearson's correlations between each gene pair to determine concordance of gene expression to generate a matrix of adjacencies, and then the adjacencies were transformed into topological overlap matrix (TOM) (Li and Horvath, 2009). Next, we performed average linkage hierarchical clustering based on the TOM-based dissimilarity with a minimum module size of 30 and a medium sensitivity of 2, and other parameters were designated as default. After relating modules to clinical traits and calculating the associated Gene Significance (the correlation between the genes and the trait) and Module Membership (the correlation of the module eigengene and the gene expression profile), we detected top 50 hub genes using a networkScreening function based on Gene Significance and Module Membership and genes with q.Weighetd value less than 0.001 were finally regarded as hub genes (Dong and Horvath, 2007).

### Functional annotation of hub genes

WebGestalt (WEB-based GEne SeT AnaLysis Toolkit) (Zhang et al., 2005; Wang et al., 2013) was used to conduct gene ontology (GO) and Kyoto Encyclopedia of Genes and Genomes (KEGG) (http://www.kegg.jp/kegg/) pathway enrichment analysis of hub genes. Search Tool for the Retrieval of Interacting Genes (STRING) database (Szklarczyk et al., 2015) was use to construct a protein-protein interaction (PPI) network, and then the PPI network was visualized using Cytoscape (Shannon et al., 2003).

### Survival analysis

Another BC microarray study GSE19915 (Lindgren et al., 2010), which included 144 BC samples and 12 normal samples and the associated clinical characteristics including gender, age, biopsy Gleason score, survival status, follow-up, etc., was regarded as a validation cohort. PROGgenesV2 (Goswami and Nakshatri, 2014) was applied to conduct log-rank based survival analyses to compare the overall survivals of particular comparing groups defined based on the medians of the expression level of hub genes. Difference with statistical significance was defined as *P* < 0.05.

### Gene set enrichment analysis (GSEA)

Bladder cancer (BC) microarray datasets GSE31684 (Riester et al., 2012, 2014) was used to conduct GSEA (Subramanian et al., 2005) analysis of hub genes. BC samples were divided into a particular hub gene high expression group and low expression group based on the median expression of this hub gene. Differences at nominal *P* < 0.05 and FDR (false discovery rate) less than 25% were defined as statistical significance.

## Results

### Results of data preprocessing, co-expression network construction and hub genes identification

**Figure 1:**
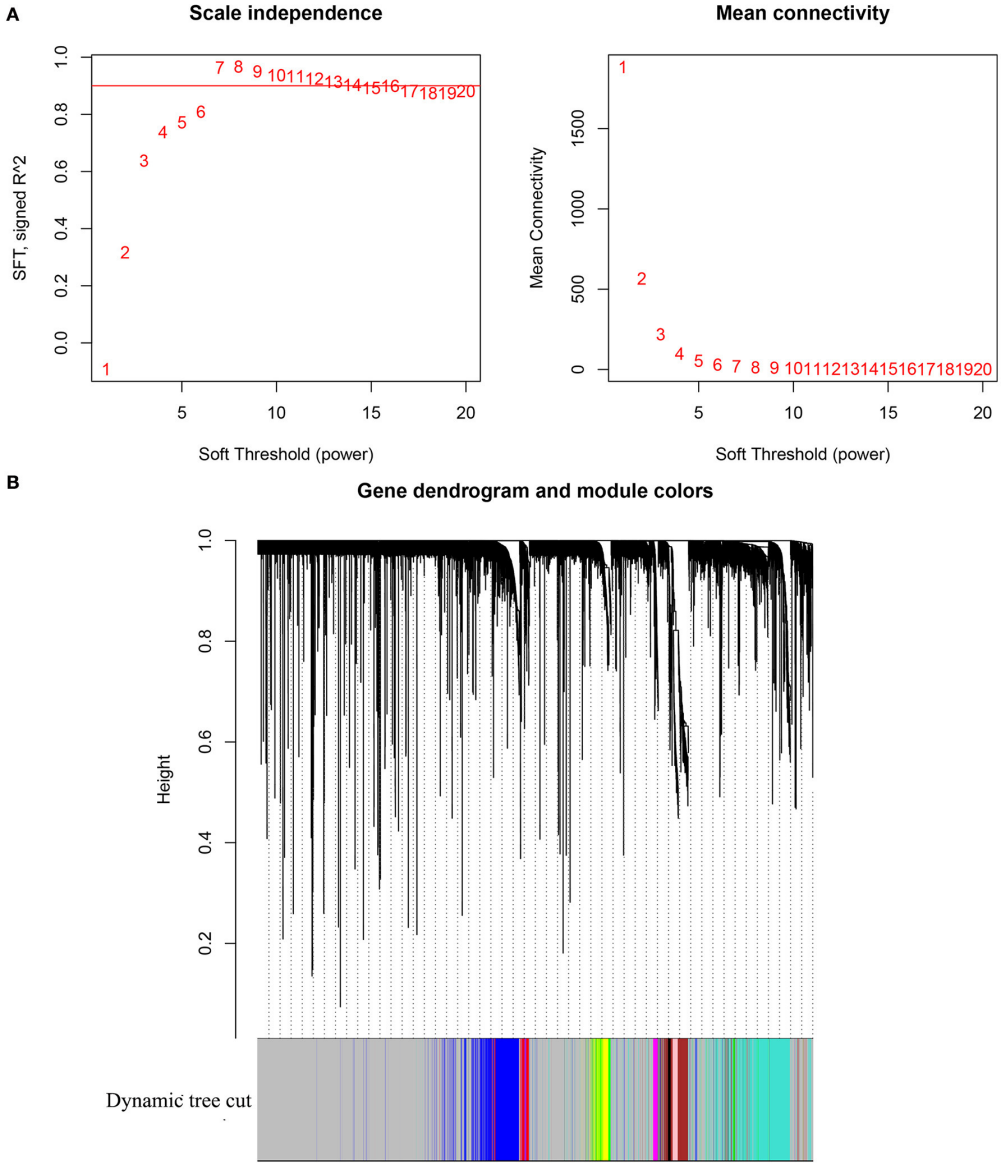
**(A)** Analysis of network topology for various soft-thresholding powers. The left panel shows the scale-free fit index (y-axis) as a function of the soft-thresholding power (x-axis). The right panel displays the mean connectivity (degree, y-axis) as a function of the soft-thresholding power (x-axis). **(B)** Clustering dendrogram of genes, with dissimilarity based on topological overlap, together with assigned module colors.

Gene expression profile of GSE13507 was obtained from GEO database, and probes with variances ranked in top 10,000 were used in the subsequent analyses. After outlier detection, one sample was excluded for further analysis (Supplementary Figure 1). As shown in Figure 1A, β = 14, the lowest power for which the scale-free topology fit index reaches 0.9, was selected for the subsequent adjacencies calculation. Based on the results of TOM based clustering, we obtained 11 gene modules as shown in Figure 1B. After the modules were related to the TNM stages of BC patients, top 50 hub genes identified based on the corresponding Gene Significance and Module Membership were summarized in Supplementary Table 1.

### Gene ontology enrichment analysis of hub genes

To get a primary understanding of the biological relevance of the hub genes, we first performed GO enrichment analysis of the hub genes. As shown in Table 1, top 10 enriched GO terms are listed. The hub genes mainly enriched in “mitotic sister chromatid segregation,” “mitotic cell cycle,” “nuclear division,” “sister chromatid segregation,” “cell cycle,” “chromosome segregation,” “mitotic nuclear division,” “cell cycle process,” “organelle fission,” and “cell division.”

**Table 1:** GO oncology analysis of top 50 hub genes.

**GO term**	***P*-Value**	**Gene symbol**
Mitotic sister chromatid segregation	<0.001	CENPA; CENPE; CENPF; KIF2C; UBE2C; CDCA5; KIF4A; NUSAP1; CDCA8; CEP55; NCAPG; BUB1B; PRC1; AURKB; KIF23; ESPL1
Mitotic cell cycle	<0.001	CDKN3; CENPA; CENPE; CENPF; KIF2C; UBE2C; CIT; CDCA5; CDCA2; FAP; TPX2; FOXM1; KIF4A; ASPM; WDR62; NUSAP1; CDCA8; CEP55; MCM10; KIF15; SPC25; NCAPG; AURKA; BUB1B; TOP2A; CDCA3; PRC1; CCNB2; AURKB; KIF23; ESPL1; MELK; CDC25B
Nuclear division	<0.001	CENPA; CENPE; CENPF; KIF2C; UBE2C; CIT; CDCA5; CDCA2; TPX2; KIF4A; ASPM; NUSAP1; CDCA8; CEP55; KIF15; SPC25; NCAPG; AURKA; BUB1B; TOP2A; CDCA3; RAD54L; PRC1; CCNB2; AURKB; KIF23; ESPL1; CDC25B
Sister chromatid segregation	<0.001	CENPA; CENPE; CENPF; KIF2C; UBE2C; CDCA5; KIF4A; NUSAP1; CDCA8; CEP55; SPC25; NCAPG; BUB1B; TOP2A; PRC1; AURKB; KIF23; ESPL1
Cell cycle	<0.001	CDKN3; CENPA; CENPE; CENPF; KIF2C; UBE2C; CIT; CDCA5; CDCA2; FAP; TPX2; FOXM1; KIF4A; ASPM; WDR62; NUSAP1; CDCA8; CEP55; MCM10; KIF15; SPC25; NCAPG; AURKA; BUB1B; TOP2A; CDCA3; RAD54L; PRC1; CCNB2; AURKB; KIF23; ESPL1; MELK; CDC25B
Chromosome segregation	<0.001	CENPA; CENPE; CENPF; KIF2C; UBE2C; CDCA5; CDCA2; KIF4A; NUSAP1; CDCA8; CEP55; SPC25; NCAPG; BUB1B; TOP2A; PRC1; AURKB; KIF23; ESPL1
Mitotic nuclear division	<0.001	CENPA; CENPE; CENPF; KIF2C; UBE2C; CIT; CDCA5; CDCA2; TPX2; KIF4A; ASPM; NUSAP1; CDCA8; CEP55; KIF15; SPC25; NCAPG; AURKA; BUB1B; CDCA3; PRC1; CCNB2; AURKB; KIF23; ESPL1; CDC25B
Cell cycle process	<0.001	CDKN3; CENPA; CENPE; CENPF; KIF2C; UBE2C; CIT; CDCA5; CDCA2; FAP; TPX2; FOXM1; KIF4A; ASPM; WDR62; NUSAP1; CDCA8; CEP55; MCM10; KIF15; SPC25; NCAPG; AURKA;BUB1B; TOP2A; CDCA3; RAD54L; PRC1; CCNB2; AURKB; KIF23; ESPL1; MELK; CDC25B
Organelle fission	<0.001	CENPA; CENPE; CENPF; KIF2C; UBE2C; CIT; CDCA5; CDCA2; TPX2; KIF4A; ASPM; NUSAP1;CDCA8; CEP55; KIF15; SPC25; NCAPG; AURKA; BUB1B; TOP2A; CDCA3; RAD54L; PRC1;CCNB2;AURKB;KIF23;ESPL1;CDC25B
Cell division	<0.001	CENPA; CENPE; CENPF; KIF2C; UBE2C; CIT; CDCA5; CDCA2; TPX2; KIF4A; ASPM; NUSAP1; CDCA8; CEP55; SPC25; NCAPG; AURKA; BUB1B; TOP2A; CDCA3; PRC1; CCNB2; AURKB; KIF23; ESPL1; CDC25B

### KEGG pathway enrichment analysis of hub genes

Moreover, as shown in Table 2, the KEGG pathway enrichment analysis of the hub genes indicated that these genes were mainly enriched in “Cell cycle,” “Oocyte meiosis,” “MicroRNAs in cancer,” “Protein digestion and absorption,” and “Progesterone-mediated oocyte maturation.”

**Table 2:** KEGG pathway analysis of top 50 hub genes.

**KEGG pathway**	***P*-value**	**Gene symbol**
Cell cycle	8.73E-05	BUB1B; CCNB2; ESPL1; CDC25B
Oocyte meiosis	0.001594	AURKA; CCNB2; ESPL1
MicroRNAs in cancer	0.003108	CDCA5; KIF23; CDC25B
Protein digestion and absorption	0.01411	COL1A1; COL5A2
Progesterone-mediated oocyte maturation	0.014713	CCNB2; CDC25B

### PPI network analysis of hub genes

**Figure 2:**
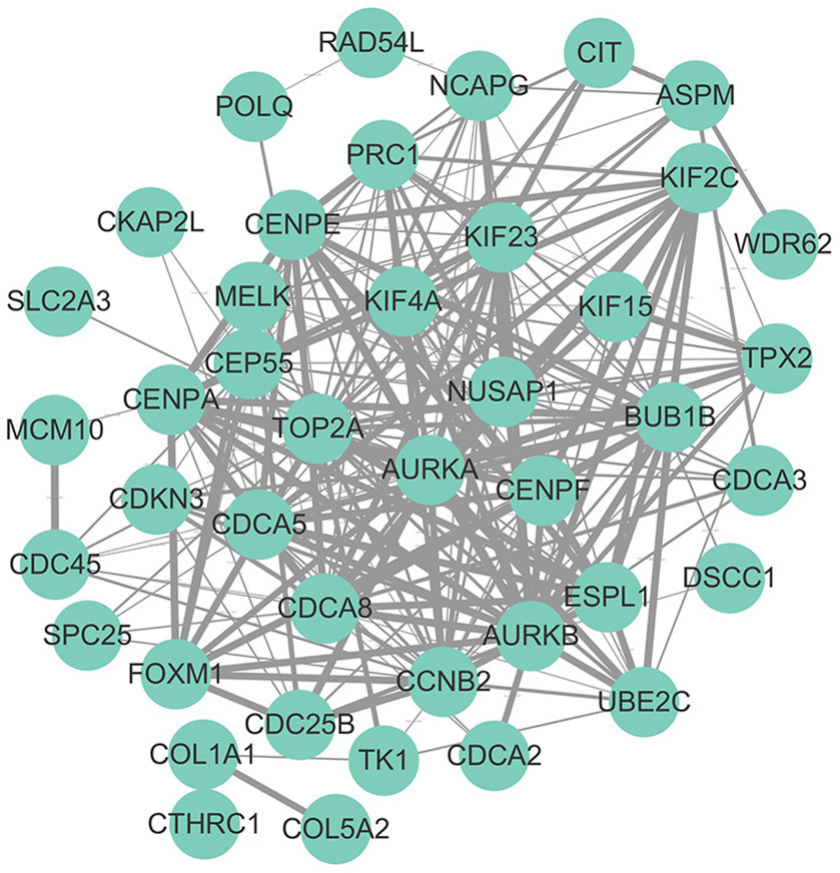
Protein-protein interaction network of hub genes. The edge width was proportional to the score of protein-protein interaction based on the STRING database.

Hub genes were mapped to STRING database. As shown in Figure 2, a total number of 244 pairs of PPIs were obtained from STRING database, and 17 hub genes (“AURKB,” “TOP2A,” “PRC1,” “KIF4A,” “CENPF,” “NUSAP1,” “KIF2C,” “CEP55,” “CDCA8,” “CENPA,” “CCNB2,” “FOXM1,” “UBE2C,” “CDC45,” “KIF15,” “BUB1B,” and “CDCA3”), interacted with ≥5 of the hub genes, were at the hub of the PPI network.

### Survival analysis of hub genes

To perform a further validation of the hub genes and identify potential biomarkers for BC, survival analyses of the hub genes were conducted using PROGgenesV2. As shown in Table 3, lower expression of 11 hub genes (including MMP11, COL5A2, CDC25B, TOP2A, CENPF, CDCA3, TK1, TPX2, CDCA8, AEBP1, and FOXM1) were significantly associated with better overall survival of BC patients.

**Table 3:** Survival of hub genes, high expression of MMP11, COL5A2, CDC25B, TOP2A, CENPF, CDCA3, TK1, TPX2, CDCA8, AEBP1, and FOXM1 were correlated with better overall survival of patients with bladder cancer.

**Gene**	**HR**	**LL**	**UL**	***P*-value**
MMP11	3.19	1.99	5.1	1.30E-06
COL5A2	2.65	1.64	4.28	6.55E-05
CDC25B	2.58	1.49	4.45	0.0006656
TOP2A	2.46	1.44	4.18	9.57E-04
CENPF	2.42	1.4	4.19	0.0015458
CDCA3	2.5	1.39	4.49	0.0021009
TK1	2.05	1.29	3.25	0.002275
TPX2	1.87	1.25	2.61	0.0024666
CDCA8	1.99	1.26	3.12	0.0029615
AEBP1	1.65	1.1	2.47	0.0148293
FOXM1	2.27	1.09	4.74	0.0293848

### GSEA analysis of hub genes that were significantly correlated with overall survival of BC patients

To characterize the potential mechanisms involved in the influences on overall survival of the above 11 hub genes. GSEA was conducted based on the expression of MMP11, COL5A2, CDC25B, TOP2A, CENPF, CDCA3, TK1, TPX2, CDCA8, AEBP1, and FOXM1. As shown in Supplementary Table 2, BC samples in MMP11 high expression group were most significantly enriched in P53 pathway; BC samples in COL5A2 high expression group were most significantly enriched in apical junction (Supplementary Table 3); BC samples in CDC25B, CENPF, TPX2, CDCA8, and FOXM1 high expression groups were most significantly enriched in mitotic spindle (Supplementary Tables 4–8); BC samples in TOP2A, CDCA3 and TK1 high expression groups were most significantly enriched in G2M checkpoint (Supplementary Tables 9–11); BC samples with AEBP1 high expression were most significantly enriched in myogenesis (Supplementary Table 12).

## Discussion

As mentioned above, BC is one of the most common cancers and significant progress has been made during the past decade. The prognosis of patients with BC, especially invasive BC, remains poor. The recurrence rate for superficial BC is about two-thirds, and, despite advances in management, some patients still develop stage progression. Meanwhile, approximately 30% of muscle-invasive BCs are associated with occult distant metastasis at the time of diagnosis, which led to a dismal 5-year survival of patient with muscle invasive BC (Kamat et al., 2016). Biomarker is defined as a substance found in tissue, blood, or other body fluids that might be a sign of cancer or noncancerous conditions (Mohammed et al., 2016). Previous studies suggested that biomarkers, including BC biomarkers, exhibited several features: prognostic, predictive, and pharmacodynamic. The biological functions of prognostic biomarker, predictive biomarker, and pharmacodynamic biomarker were predicting the natural course of cancers, evaluating the probable benefit of a particular treatment, and assessing the treatment effects of a drug on a tumor and determine the proper dosage of a new anticancer drug, respectively (Kojima et al., 2016; Mohammed et al., 2016; Nandagopal and Sonpavde, 2016). Nowadays, many biomarkers and their corresponding targeted agents have been determined for the diagnosis and treatment of BC. In the present study, we identified 50 hub genes that were significantly correlated with the TNM staging of BC patients using WGCNA.

As we know, TNM staging, devised by Pierre Denoix, describes the size of the original (primary) tumor and whether it has invaded nearby tissue (T), describes nearby (regional) lymph nodes that are involved (N), and describes distant metastasis (M) (Denoix, 1946). The functional enrichment analysis of hub genes that were correlated with the TNM stages indicated that these genes were enriched in cell proliferation associated GO terms (mitotic sister chromatid segregation, mitotic cell cycle and cell cycle, etc.) and KEGG pathways (cell cycle, Oocyte meiosis, etc.). Thus, we speculated that these hub genes affected the TNM staging of BC patients through promoting the proliferation of BC cells.

The PPI network analysis of the hub genes suggested that a total number of 17 hub genes interacted with ≥5 of the hub genes were at the hub of the PPI network. Previous studies demonstrated that functional partnerships and interactions that occurred between proteins were at the core of cellular processing and their systematic characterization helped to provide context in molecular systems biology. Thus, the 17 hub genes, as mentioned above, might play an important role in the biological process of BC cells.

Our survival analyses of the hub genes indicated that lower expressions of 11 hub genes were correlated with relatively shorter overall survivals of patients with BC. Meanwhile, our GSEA results indicated that BC samples with higher expression of these genes were enriched in genes sets that were correlated with biological behaviors of tumor cells (such as P53 pathway, Apical junction, mitotic spindle, G2M checkpoint, Myogenesis, etc.).

As for the 11 survival associated hub genes, we conducted a literature review of these genes. MMP11 is a member of the matrix metalloproteinase (MMP) family involved in the breakdown of extracellular matrix in normal physiological processes, such as embryonic development, reproduction, and tissue remodeling, as well as in disease processes, and several studies demonstrated that overexpression of MMP11 were correlated with many cancers (including colon cancer, laryngeal cancer, breast cancer, prostate cancer, etc.) (Deng et al., 2005; Kou et al., 2013; Roscilli et al., 2014; Li et al., 2015; Pang et al., 2016); COL5A2 encodes an alpha chain for one of the low abundance fibrillar collagens, and previous studies suggested that COL5A2 was associated with malignancy in colorectal cancer, breast cancer, osteosarcoma, etc. (Fischer et al., 2001; Vargas et al., 2012; Wu et al., 2014); CDC25B is a member of the CDC25 family of phosphatases, and previous studies suggested that elevated expression of CDC25B promoted the proliferation of gastric cancer, esophageal carcinoma, renal cell carcinoma, etc. (Yu et al., 2012; Wang et al., 2015; Leal et al., 2016); TOP2A encodes a DNA topoisomerase, and previous studies suggested that TOP2A overexpression was a poor prognostic factor in patients with breast cancer, colorectal cancer, prostate cancer, and nasopharyngeal carcinoma, etc., (de Resende et al., 2013; Lan et al., 2014; Tarpgaard et al., 2016; Zheng et al., 2016); CENPF is a component of the nuclear matrix during the G2 phase of interphase, and previous studies demonstrated that CENPF was associated with the tumor progression of patients with prostate cancer, hepatocellular carcinomas, and breast cancer, etc. (Brendle et al., 2009; Kim et al., 2012); Cell division cycle associated 3 (CDCA3) is a part of the Skp1-cullin-F-box (SCF) ubiquitin ligase and refers to a trigger of mitotic entry and mediates destruction of the mitosis inhibitory kinase, and previous studies demonstrated overexpression of CDCA3 promoted progression of several cancers (oral cancer, prostate cancer, and lung cancer, etc.) (Uchida et al., 2012; Chen et al., 2013; O'Byrne et al., 2016); TK1(thymidine kinase 1) encodes a cytosolic enzyme that catalyzes the addition of a gamma-phosphate group to thymidine, and previous studies proved that high TK1 expression is a predictor of poor survival in patients with pT1 of lung adenocarcinoma, BC, gastrointestinal cancer, etc. (Jagarlamudi et al., 2015; Du et al., 2016); Targeting protein for Xenopus kinesin-like protein 2 (TPX2) is microtubule-associated protein and impacts spindle assembly in human cells (Huang et al., 2014), and previous studies proved that TPX2 expression was associated with poor survival in gastric cancer, breast cancer, colon cancer and esophageal cancer, etc. (Wei et al., 2013; Liu et al., 2014; Yang et al., 2015; Tomii et al., 2017); The cell division cycle associated 8 (CDCA8) gene encodes a component of the chromosomal passenger complex and plays an important role in mitosis, and overexpression of CDCA8 was reported in some human cancers, demonstrating that CDCA8 was required for the growth and progression of several cancers such as breast cancer, gastric cancer, lung carcinogenesis (Hayama et al., 2007; Yan et al., 2012; Jiao et al., 2015); Several studies demonstrated the AEBP1 upregulation conferred acquired resistance to BRAF (V600E) inhibition in melanoma and was associated with bad survivals of patients with glioma (Ladha et al., 2012; Hu et al., 2013); The protein encoded by FOXM1 is phosphorylated in M phase and regulates the expression of several cell cycle genes, such as cyclin B1 and cyclin D1, and previous studies suggested that FOXM1 promoted tumor progression of multiple cancers including gastric cancer, ovarian cancer, cervical cancer, colorectal cancer and breast cancer, etc. Trichostatin A potentiates TRAIL-induced antitumor effects via inhibition of ERK/FOXM1 pathway in gastric cancer (Barger et al., 2015; Yau et al., 2015; Zheng et al., 2015; Li et al., 2016; Wang et al., 2016; Song et al., 2017). In summary, all the conclusions were consistent with the results of survival analysis and GSEA analysis that high expressions of MMP11, COL5A2, CDC25B, TOP2A, CENPF, CDCA3, TK1, TPX2, CDCA8, AEBP1, and FOXM1 were correlated with worse overall survival of BC patients and BC samples with relatively higher expression of these genes were enriched in gene sets that were associated with cell proliferation.

In conclusion, the identified 50 hub genes that were closely correlated with the TNM staging of BC patients, and 11 hub genes (MMP11, COL5A2, CDC25B, TOP2A, CENPF, CDCA3, TK1, TPX2, CDCA8, AEBP1, and FOXM1) of which were significantly correlated with the overall survival of BC, which could be candidate biomarkers for BC. Meanwhile, further *in vivo* and *in vitro* studies were needed to make clear the exact molecular mechanisms that affected the growth of BC cells.

## Author contributions

XW designed the study. SL and XL collected, analyzed and interpreted the data. XL, TL, XM, XY, CF, DH, and HW participated in revising the manuscript. XZ and YC participated in the study design and helped to draft the manuscript. All authors read and approved the final manuscript.

### Conflict of interest statement

The authors declare that the research was conducted in the absence of any commercial or financial relationships that could be construed as a potential conflict of interest.

## Supplementary material

Supplementary Figure 1 Cluster tree of bladder cancer samples. The leaves of the tree correspond to the bladder cancer sample. The first color band underneath the tree indicates which arrays appear to be outlying. The sixth color band represents TNM staging (red indicates high values). Similarly, the remaining color-bands color-code the numeric values of physiologic traits.
