# Supplementary figures and images for: Identification of Biomarkers Correlated with the TNM Staging and Overall Survival of Patients with Bladder Cancer
Source: Front Physiol. 2017 Nov 28;8:947. doi: 10.3389/fphys.2017.00947 (PMC5712410; doi:10.3389/fphys.2017.00947)

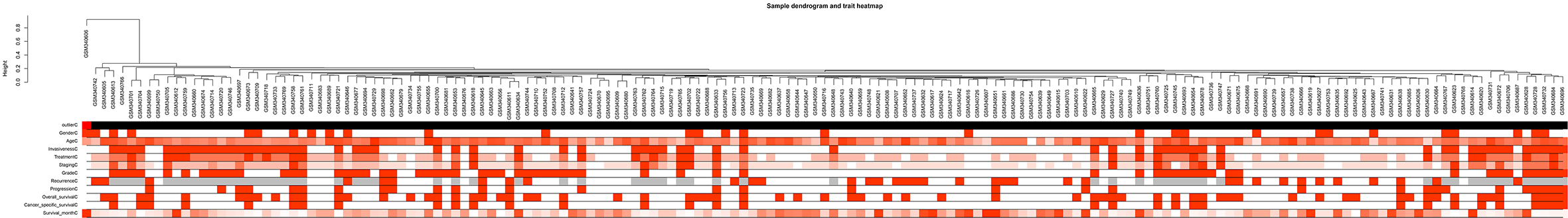

Supplement: Supplementary Figure 1 — Cluster tree of bladder cancer samples. The leaves of the tree correspond to the bladder cancer sample. The first color band underneath the tree indicates which arrays appear to be outlying. The sixth color band represents TNM staging (red indicates high values). Similarly, the remaining color-bands color-code the numeric values of physiologic traits. [file Image1.TIF]
