# Supplementary material for: Identification of Biomarkers Correlated with the TNM Staging and Overall Survival of Patients with Bladder Cancer
Source: Front Physiol. 2017 Nov 28;8:947. doi: 10.3389/fphys.2017.00947 (PMC5712410; doi:10.3389/fphys.2017.00947)
Supplement: Supplementary file 2 [file SupplementaryTables1-12.docx]

**Supplementary table 1** Top 50 hub genes

| Symbol | q.Weighted | cor.Weighted | cor.Standard |
| --- | --- | --- | --- |
| COL5A2 | 0 | 0.837077 | 0.521385 |
| FAP | 0 | 0.749499 | 0.451129 |
| COL1A1 | 0 | 0.736114 | 0.449839 |
| KIF2C | 0 | 0.72338 | 0.348309 |
| DCC1 | 0 | 0.716041 | 0.363311 |
| CTHRC1 | 0 | 0.715462 | 0.435081 |
| CEP55 | 0 | 0.715166 | 0.324642 |
| C15orf48 | 0 | 0.714414 | 0.417136 |
| CDCA5 | 0 | 0.714065 | 0.334171 |
| WDR62 | 0 | 0.706427 | 0.427706 |
| BUB1B | 0 | 0.706092 | 0.304226 |
| CCNB2 | 0 | 0.705606 | 0.30769 |
| POLQ | 0 | 0.702788 | 0.384904 |
| CENPA | 0 | 0.701031 | 0.295302 |
| MMP11 | 0 | 0.698085 | 0.454831 |
| CENPF | 0 | 0.697587 | 0.345725 |
| CKAP2L | 0 | 0.6974 | 0.333761 |
| CIT | 0 | 0.692416 | 0.370093 |
| SLC2A3 | 0 | 0.691528 | 0.402532 |
| TPX2 | 0 | 0.689692 | 0.348481 |
| MCM10 | 0 | 0.688553 | 0.284023 |
| RAD54L | 0 | 0.686804 | 0.346767 |
| AEBP1 | 0 | 0.685954 | 0.379738 |
| CENPE | 0 | 0.682084 | 0.316804 |
| TK1 | 0 | 0.681036 | 0.321104 |
| CDKN3 | 0 | 0.678133 | 0.299977 |
| AURKA | 0 | 0.674062 | 0.295639 |
| PRC1 | 0 | 0.671757 | 0.297961 |
| NCAPG | 0 | 0.671527 | 0.335671 |
| ASPM | 0 | 0.670925 | 0.336667 |
| TOP2A | 0 | 0.670354 | 0.29839 |
| NUSAP1 | 0 | 0.6674 | 0.317309 |
| LILRB3 | 0 | 0.666203 | 0.362055 |
| MELK | 0 | 0.665487 | 0.287335 |
| CDC45L | 0 | 0.662509 | 0.304993 |
| CDCA8 | 0 | 0.661737 | 0.287291 |
| C17orf53 | 0 | 0.660045 | 0.377919 |
| FAM20C | 0 | 0.653991 | 0.337736 |
| C1orf135 | 0 | 0.653883 | 0.304036 |
| ESPL1 | 0 | 0.651365 | 0.323969 |
| KIF23 | 0 | 0.650513 | 0.286883 |
| KIF4A | 0 | 0.646767 | 0.243748 |
| KIF15 | 0 | 0.64665 | 0.285735 |
| SPC25 | 0 | 0.645363 | 0.294918 |
| UBE2C | 0 | 0.645248 | 0.285606 |
| CDCA3 | 0 | 0.64506 | 0.262539 |
| AURKB | 0 | 0.643783 | 0.310402 |
| CDC25B | 0 | 0.642307 | 0.405433 |
| FOXM1 | 0 | 0.641813 | 0.311869 |
| CDCA2 | 0 | 0.641277 | 0.294356 |

**Supplementary table 2** Gene set enriched in bladder samples with MMP11 high expression

| MMP11 | ES | NES | NOM p-val | FDR q-val |
| --- | --- | --- | --- | --- |
| P53 pathway | 0.569975 | 1.748534 | 0.001953 | 0.053977 |
| Glycolysis | 0.509253 | 1.670371 | 0.001992 | 0.051434 |
| Hypoxia | 0.58571 | 1.712101 | 0.003968 | 0.049811 |
| TNF α signaling via NF -κ B | 0.706947 | 1.686971 | 0.008214 | 0.049834 |
| Estrogen response late | 0.453254 | 1.402984 | 0.01996 | 0.235255 |
| Apoptosis | 0.539821 | 1.558617 | 0.028689 | 0.157919 |
| Coagulation | 0.591051 | 1.548977 | 0.031315 | 0.125687 |
| Apical surface | 0.51761 | 1.450372 | 0.03629 | 0.181171 |
| Hedgehog signaling | 0.570522 | 1.503859 | 0.041322 | 0.164895 |

ES, enrichment score; NES, normalized enrichment score; NOM p-val, nominal p value; FDR, false discovery rate q value.

**Supplementary table 3** Gene set enriched in bladder samples with COL5A2 high expression

| COL5A2 | ES | NES | NOM p-val | FDR q-val |
| --- | --- | --- | --- | --- |
| Apical junction | 0.627079 | 1.889416 | 0 | 0.002013 |
| Coagulation | 0.71376 | 1.868791 | 0 | 0.001507 |
| Epithelial mesenchymal  transition | 0.871183 | 1.856636 | 0 | 0.002696 |
| Hypoxia | 0.640134 | 1.847487 | 0 | 0.00223 |
| Ultraviolet response | 0.635863 | 1.843251 | 0 | 0.001784 |
| Angiogenesis | 0.790621 | 1.822863 | 0 | 0.002105 |
| Apoptosis | 0.611613 | 1.782093 | 0 | 0.005605 |
| KRAS signaling up | 0.702134 | 1.768691 | 0 | 0.005683 |
| Complement | 0.671026 | 1.756269 | 0 | 0.005821 |
| Inflammatory response | 0.719478 | 1.724852 | 0 | 0.007976 |
| Hedgehog signaling | 0.632732 | 1.671999 | 0.002016 | 0.014508 |
| Myogenesis | 0.618571 | 1.743892 | 0.002058 | 0.006697 |
| IL2-STAT5 signaling | 0.588156 | 1.605062 | 0.002232 | 0.0319 |
| IL6-JAK - STAT3 signaling | 0.726148 | 1.723509 | 0.004141 | 0.007362 |
| TNF α signaling via NF -κ B | 0.716782 | 1.741004 | 0.006073 | 0.006391 |
| Apical surface | 0.547868 | 1.528041 | 0.007921 | 0.05667 |
| Notch signaling | 0.603928 | 1.586561 | 0.012245 | 0.035903 |
| Allograft rejection | 0.746026 | 1.57427 | 0.023158 | 0.037805 |
| Glycolysis | 0.439276 | 1.454611 | 0.036585 | 0.086799 |
| Androgen response | 0.441273 | 1.435938 | 0.038618 | 0.094875 |

ES, enrichment score; NES, normalized enrichment score; NOM p-val, nominal p value; FDR, false discovery rate q value.

**Supplementary table 4** Gene set enriched in bladder samples with CDC25B high expression

| CDC25B | ES | NES | NOM p-val | FDR q-val |
| --- | --- | --- | --- | --- |
| Mitotic spindle | 0.537299 | 1.725781 | 0.001988 | 0.118782 |
| Spermatogenesis | 0.49028 | 1.559072 | 0.005988 | 0.159185 |
| Hedgehog signaling | 0.591607 | 1.60269 | 0.009452 | 0.162495 |
| Allograft rejection | 0.755676 | 1.567714 | 0.011673 | 0.183708 |
| G2M checkpoint | 0.62833 | 1.639232 | 0.013807 | 0.159919 |
| Pancreas beta cells | 0.633365 | 1.520905 | 0.028571 | 0.163874 |
| Apical junction | 0.484647 | 1.440308 | 0.037328 | 0.129648 |
| E2F targets | 0.651118 | 1.529277 | 0.037849 | 0.175013 |

ES, enrichment score; NES, normalized enrichment score; NOM p-val, nominal p value; FDR, false discovery rate q value.

**Supplementary table 5** Gene set enriched in bladder samples with CENPF high expression

| CENPF | ES | NES | NOM p-val | FDR q-val |
| --- | --- | --- | --- | --- |
| Mitotic spindle | 0.609129 | 2.002083 | 0 | 0 |
| G2M checkpoint | 0.756378 | 1.957191 | 0 | 0 |
| E2F targets | 0.795231 | 1.84273 | 0 | 0.002499 |
| Spermatogenesis | 0.524928 | 1.689393 | 0 | 0.010732 |
| MYC targets | 0.634715 | 1.78841 | 0.002024 | 0.005644 |
| DNA repair | 0.508607 | 1.704911 | 0.013672 | 0.012034 |
| mTORC1 signaling | 0.511922 | 1.67723 | 0.022222 | 0.011703 |

ES, enrichment score; NES, normalized enrichment score; NOM p-val, nominal p value; FDR, false discovery rate q value.

**Supplementary table 6** Gene set enriched in bladder samples with TPX2 high expression

| TPX2 | ES | NES | NOM p-val | FDR q-val |
| --- | --- | --- | --- | --- |
| Mitotic spindle | 0.590704 | 1.911951 | 0 | 0.001743 |
| G2M checkpoint | 0.717889 | 1.820862 | 0 | 0.004462 |
| Spermatogenesis | 0.55879 | 1.778542 | 0 | 0.008041 |
| E2F targets | 0.76323 | 1.748894 | 0 | 0.007729 |
| mTORC1 signaling | 0.542369 | 1.744517 | 0.001852 | 0.006749 |
| MYC targets | 0.618844 | 1.764144 | 0.005747 | 0.00715 |
| DNA repair | 0.484858 | 1.618072 | 0.028846 | 0.04992 |
| Unfolded protein response | 0.457019 | 1.53746 | 0.031189 | 0.109479 |

ES, enrichment score; NES, normalized enrichment score; NOM p-val, nominal p value; FDR, false discovery rate q value.

**Supplementary table 7** Gene set enriched in bladder samples with CDCA8 high expression

| CDCA8 | ES | NES | NOM p-val | FDR q-val |
| --- | --- | --- | --- | --- |
| Mitotic spindle | 0.582269 | 1.875646 | 0 | 0.00344 |
| Spermatogenesis | 0.561117 | 1.82465 | 0 | 0.004577 |
| G2M checkpoint | 0.708252 | 1.844095 | 0.002016 | 0.004407 |
| E2F targets | 0.764565 | 1.768998 | 0.00202 | 0.0091 |
| MYC targets | 0.601324 | 1.691518 | 0.004065 | 0.023855 |
| Unfolded protein response | 0.511365 | 1.759133 | 0.006122 | 0.008204 |
| Ultraviolet response | 0.431717 | 1.477603 | 0.007828 | 0.144549 |
| mTORC1 signaling | 0.530754 | 1.663194 | 0.010482 | 0.030199 |
| DNA repair | 0.481746 | 1.606822 | 0.014286 | 0.054924 |
| Pancreas beta cells | 0.648692 | 1.538302 | 0.02004 | 0.099006 |

ES, enrichment score; NES, normalized enrichment score; NOM p-val, nominal p value; FDR, false discovery rate q value.

**Supplementary table 8** Gene set enriched in bladder samples with FOXM1 high expression

| FOXM1 | ES | NES | NOM p-val | FDR q-val |
| --- | --- | --- | --- | --- |
| Mitotic spindle | 0.582269 | 1.85528 | 0 | 0.004048 |
| G2M checkpoint | 0.708252 | 1.786583 | 0 | 0.013796 |
| Spermatogenesis | 0.561117 | 1.770968 | 0 | 0.010462 |
| E2F targets | 0.764565 | 1.742011 | 0 | 0.012355 |
| Unfolded protein response | 0.511365 | 1.733983 | 0 | 0.011118 |
| MYC targets | 0.601324 | 1.681728 | 0.001835 | 0.020141 |
| mTORC1 signaling | 0.530754 | 1.69683 | 0.009728 | 0.01725 |
| Pancreas beta cells | 0.648692 | 1.578798 | 0.015474 | 0.061694 |
| Ultraviolet response | 0.431717 | 1.447619 | 0.017647 | 0.193483 |
| DNA repair | 0.481746 | 1.592241 | 0.026415 | 0.061235 |

ES, enrichment score; NES, normalized enrichment score; NOM p-val, nominal p value; FDR, false discovery rate q value.

**Supplementary table 9**  Gene set enriched in bladder samples with TOP2A high expression

| TOP2A | ES | NES | NOM p-val | FDR q-val |
| --- | --- | --- | --- | --- |
| G2M checkpoint | -0.73642 | -1.91964 | 0 | 0 |
| E2F targets | -0.80723 | -1.87579 | 0 | 5.01E-04 |
| Mitotic spindle | -0.58919 | -1.86804 | 0 | 3.34E-04 |
| MYC targets | -0.6366 | -1.81412 | 0 | 0.002509 |
| Spermatogenesis | -0.53576 | -1.71074 | 0 | 0.011333 |
| DNA repair | -0.50834 | -1.70587 | 0.005871 | 0.010444 |
| Mtorc1 signaling | -0.51057 | -1.61574 | 0.023166 | 0.027215 |

ES, enrichment score; NES, normalized enrichment score; NOM p-val, nominal p value; FDR, false discovery rate q value.

**Supplementary table 10** Gene set enriched in bladder samples with CDCA3 high expression

| CDCA3 | ES | NES | NOM p-val | FDR q-val |
| --- | --- | --- | --- | --- |
| G2M checkpoint | 0.732191 | 1.895866 | 0 | 0.004717 |
| E2F targets | 0.787671 | 1.844714 | 0 | 0.005975 |
| Spermatogenesis | 0.524459 | 1.69725 | 0 | 0.023369 |
| Mitotic spindle | 0.534079 | 1.752156 | 0.002066 | 0.013245 |
| MYC targets | 0.642537 | 1.916296 | 0.005917 | 0.007844 |
| mTORC1 signaling | 0.51422 | 1.672505 | 0.013216 | 0.024449 |
| DNA repair | 0.488797 | 1.674041 | 0.013752 | 0.026985 |
| Unfolded protein response | 0.448311 | 1.582801 | 0.038776 | 0.066773 |
| Glycolysis | 0.426582 | 1.413847 | 0.038793 | 0.205351 |

ES, enrichment score; NES, normalized enrichment score; NOM p-val, nominal p value; FDR, false discovery rate q value.

**Supplementary table 11** Gene set enriched in bladder samples with TK1 high expression

| TK1 | ES | NES | NOM p-val | FDR q-val |
| --- | --- | --- | --- | --- |
| G2M checkpoint | -0.7308 | -1.90589 | 0 | 1.00E-03 |
| mTORC1 signaling | -0.5959 | -1.88546 | 0 | 5.00E-04 |
| E2F targets | -0.78076 | -1.84781 | 0 | 0.001924 |
| Reactive oxygen species  pathway | -0.5736 | -1.72003 | 0.002004 | 0.014683 |
| DNA repair | -0.52902 | -1.77892 | 0.00202 | 0.006185 |
| Myc targets | -0.63121 | -1.79906 | 0.002062 | 0.003639 |
| Glycolysis | -0.47684 | -1.55894 | 0.005882 | 0.075504 |
| Spermatogenesis | -0.47331 | -1.54872 | 0.008584 | 0.074501 |
| Mitotic spindle | -0.51624 | -1.67495 | 0.016495 | 0.026557 |
| Unfolded protein response | -0.47943 | -1.64145 | 0.027833 | 0.034422 |
| Oxidative phosphorylation | -0.52057 | -1.67095 | 0.037109 | 0.024328 |

ES, enrichment score; NES, normalized enrichment score; NOM p-val, nominal p value; FDR, false discovery rate q value.

**Supplementary table 12** Gene set enriched in bladder samples with AEBP1 high expression

| AEBP1 | ES | NES | NOM p-val | FDR q-val |
| --- | --- | --- | --- | --- |
| Myogenesis | -0.66328 | -1.88658 | 0 | 0.006315 |
| Apical junction | -0.62727 | -1.84594 | 0 | 0.007691 |
| Coagulation | -0.71413 | -1.83741 | 0 | 0.005839 |
| Epithelial mesenchymal  _transition | -0.84443 | -1.78626 | 0 | 0.006822 |
| KRAS signaling | -0.6977 | -1.78253 | 0 | 0.005903 |
| Apoptosis | -0.61714 | -1.77233 | 0 | 0.005424 |
| Ultraviolet response | -0.61309 | -1.73895 | 0 | 0.006618 |
| Hypoxia | -0.59544 | -1.72006 | 0 | 0.00923 |
| Complement | -0.64208 | -1.6804 | 0.004008 | 0.013507 |
| Angiogenesis | -0.72384 | -1.69004 | 0.004057 | 0.013277 |
| IL6-JAK - STAT3 signaling | -0.70196 | -1.64739 | 0.004107 | 0.021661 |
| Notch signaling | -0.66399 | -1.74534 | 0.004158 | 0.006968 |
| IL2- STAT5 signaling | -0.574 | -1.5906 | 0.006316 | 0.037187 |
| Inflammatory response | -0.68645 | -1.61163 | 0.008511 | 0.03139 |
| Apical surface | -0.53308 | -1.45822 | 0.020747 | 0.098776 |
| Allograft rejection | -0.74354 | -1.55779 | 0.029661 | 0.049804 |
| TNF α signaling via NF -κ B | -0.63586 | -1.5176 | 0.039583 | 0.067517 |
| Hedgehog signaling | -0.55634 | -1.444 | 0.044088 | 0.104563 |
| Interferon gamma response | -0.71458 | -1.49617 | 0.048421 | 0.078202 |

ES, enrichment score; NES, normalized enrichment score; NOM p-val, nominal p value; FDR, false discovery rate q value.
